# Incidence and risk factors for surgical site infection in general surgeries ^1^

**DOI:** 10.1590/1518-8345.1502.2848

**Published:** 2017-12-04

**Authors:** Rafael Lima Rodrigues de Carvalho, Camila Cláudia Campos, Lúcia Maciel de Castro Franco, Adelaide De Mattia Rocha, Flávia Falci Ercole

**Affiliations:** 2 Doctoral student, Escola de Enfermagem, Universidade Federal de Minas Gerais, Belo Horizonte, MG, Brasil. Professor, Escola de Enfermagem, Universidade Federal de Minas Gerais, Belo Horizonte, MG, Brazil.; 3 Doctoral student, Escola de Enfermagem, Universidade Federal de Minas Gerais, Belo Horizonte, MG, Brazil.; 4 PhD, Associate Professor, Escola de Enfermagem, Universidade Federal de Minas Gerais, Belo Horizonte, MG, Brazil.

**Keywords:** Incidence, Surgical Wound Infection, Risk Factors, Epidemiological Surveillance, General Surgery, Nursing

## Abstract

**Objective:**

to estimate the incidence of surgical site infection in general surgeries at a large Brazilian hospital while identifying risk factors and prevalent microorganisms.

**Method:**

non-concurrent cohort study with 16,882 information of patients undergoing general surgery from 2008 to 2011. Data were analyzed by descriptive, bivariate and multivariate analysis.

**Results:**

the incidence of surgical site infection was 3.4%. The risk factors associated with surgical site infection were: length of preoperative hospital stay more than 24 hours; duration of surgery in hours; wound class clean-contaminated, contaminated and dirty/infected; and ASA index classified into ASA II, III and IV/V. *Staphyloccocus aureus* and *Escherichia coli* were identified.

**Conclusion:**

the incidence was lower than that found in the national studies on general surgeries. These risk factors corroborate those presented by the National Nosocomial Infection Surveillance System Risk Index, by the addition of the length of preoperative hospital stay. The identification of the actual incidence of surgical site infection in general surgeries and associated risk factors may support the actions of the health team in order to minimize the complications caused by surgical site infection.

## Introduction

Healthcare-Associated Infections (HAIs) is a subject of great concern of the healthcare services. Among the topographies of the HAIs, Surgical Site Infection (SSI) is directly related to surgical procedures, and is currently one of the most important among the HAIs^1^ ^-^ ^3^.

In a study of the *National Healthcare Safety Network* (NHSN) involving information of 850,000 general surgeries performed in the United States, it was found an overall incidence of SSI equal to 1.9%^2^. In Brazil, data on the incidence of SSI in general and specific surgeries vary from 1.4% to 38.8%^3^ ^-^ ^9^. It is important to note that, of these studies, only two refer to data from general surgeries^3^ ^,^ ^8^.

SSI leads to serious consequences, including increased costs due to its treatment^10^ and increased length of hospital stay^10^ ^-^ ^11^. The risk of death in patients with SSI is increased when compared to those who did not develop an infection^11^. 

The serious consequences imposed on patients who developed SSI determine the need for efforts to create strategies for the prevention of this infection. One of the strategies used is the determination of risk factors, which allows identifying clinical situations or conditions that predispose to the development of SSI. In this sense, the identification of risk factors for SSI contributes to the early adoption of nursing interventions that aim to minimize this type of postoperative complication.

Several risk factors are known in the literature as predisposing to SSI and make up the surgical infection risk index of the *National Nosocomial Infection Surveillance System* (NNIS)^12^, such as the *American Society of Anesthesiologists* (ASA) index, which classifies patients according to their clinical condition^1^; the Wound class, which represents the classification of the surgical wound by the surgical team in terms of the potential presence of microorganisms^1^ and; the Duration of Surgery^4^ ^,^ ^13^. 

 Other risk factors such as: Body Mass Index (BMI)^13^ ^),^ smoking^5^, video-assisted procedures^13^ ^-^ ^4^, blood transfusion^9^, non-performance of preoperative bath^9^ and pre-existing chronic diseases^1^ ^,^ ^9^ ^,^ ^13^, are also mentioned in the literature and were identified as associated with SSI, in studies on the subject. 

In the Brazilian literature, there is a lack of studies on general surgeries, which makes it difficult to estimate the SSI rates and the identification of risk factors associated with infection. Therefore this study arose from the need to identify risk factors for SSI in general surgeries, since the scientific production on this subject has privileged the survey in specific surgeries^4^ ^-^ ^7^ ^,^ ^9^. 

This study aimed to estimate the incidence of surgical site infection in general surgeries at a large Brazilian hospital while identifying risk factors and prevalent microorganisms.

## Method

This is a non-concurrent cohort study, performed in a large general hospital in Belo Horizonte, from January 2008 to December 2011.

The study hospital offers highly complex hospital and ambulatory care, with a capacity for 516 beds and an average of 582 surgical interventions of several specialties per month. It has a Hospital Infection Control Service (HICS), which is composed of a team that performs infection surveillance according to the NHSN methodology 2008^12^. 

It should be noted that, the definition of SSI used for the diagnosis of infection by the medical team responsible for patient follow-up during hospitalization is the NHSN definition. This definition considers as infection that occurring within 30 days after an NHSN surgical procedure or up to one year in case of implant use and can affect skin, tissue and organ and space^12^. 

All information on surgeries and SSI, as well as data on microbiological cultures, were collected by the HICS staff members through active search and consultation on medical charts during the hospitalization of the patients. This information was stored in the database of the Computerized Hospital Infection Control Service (SACIH) of the hospital’s HICS.

Data extracted from SACIH software were entered into an EXCEL spreadsheet by the researchers, and then exported to STATA 12 software for statistical analysis (StataCorp, College Station, TX). Access to the SACIH database was authorized by the study hospital administration and by the coordination of the HICS.

As inclusion criterion, information was selected from patients submitted to general surgeries classified as NHSN and performed in patients older than 18 years. An NHSN procedure is defined as that performed in an operating room where the surgeon makes at least one incision, which is closed before leaving the operating room.

**Figure 1:**
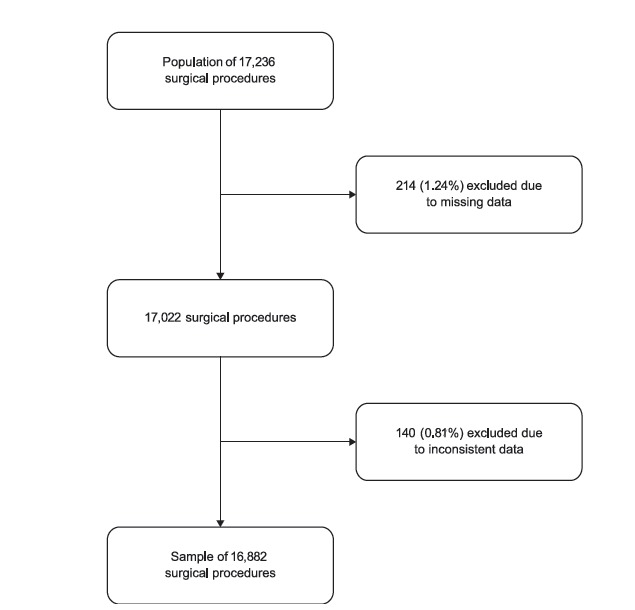
Diagram of the methodological flow of the study, Belo Horizonte, MG, Brazil, 2011

Initially, the database had information on 20,124 general surgical procedures. After applying the inclusion criteria, a population of 17,236 procedures was achieved. In analyzing the data consistency, the missing and/or inconsistent information identified in each database variable were excluded and data were analyzed in relation to the complete information in order to verify the occurrence or not of the differential loss. It should be noted that study losses were classified as non-differential. Therefore, a final sample of 16,882 procedures was used (Figure 1).

The presence or absence of SSI was considered as a dependent variable. The following independent variables were analyzed: gender (male and female); age (under and above 54 years old, according to the average, since the variable presents normal distribution); preoperative hospital stay (greater and less than 24 hours before the surgical procedure, as recommended by the National Health Surveillance Agency - ANVISA); duration of surgery (in hours); PCSW (clean, clean-contaminated, contaminated or dirty/infected); the ASA index (ASA I, ASA II, ASA III or ASA IV/V); emergency surgery (yes and no); use of general anesthesia (yes and no) and implant use (yes and no). The variables age and length of preoperative hospital stay were collected as continuous variables and subsequently dichotomized. The variable duration of surgery was collected and analyzed as a continuous variable.

In the descriptive analysis of the data, simple frequency, central tendency measurement (mean and median) and measures of variability (standard deviation) were used.

The overall incidence of SSI was calculated for the study period. Logistic regression model was used to analyze the association between the independent variables with SSI, with a significance level set at 20%. In multivariate analysis, the selected variables were removed one by one, according to the *stepwise backward* method*,* considering a “p-value” less than 0.05 and the *Log Likelihood Ratio* (LLR), indicating the contribution of the variable the best adjustment of the model.

The Research Ethics Committee of the Federal University of Minas Gerais approved this study (ETIC 14504413.1.0000.5149).

## Results

Of the 16,882 surgical procedures, 11,897 (70.5%) were performed in female patients. The mean age was 54.2 years ± 16.4 (18-99), with a median of 55 years. The mean duration of surgeries was 1.6 hours ± 1.0 (0.2-20.9), with a median of 1.2 hours.

During the study period, 568 SSI were diagnosed, with a global incidence of 3.4% [95% CI = 3.1 - 3.6] among all procedures (16,882).

Bivariate analysis showed an association between most of the independent variables with the dependent variable SSI (p<0.20), except for age and implant use (Table 1).

**Table 1:** Bivariate analysis of the independent covariates in relation to Surgical Site Infection. Belo Horizonte, MG, Brazil, 2008-2011

**Variables**		**Surgical site infection**				OR*		**95% CI** ^**†**^	**P value**
		**No**			**Yes**				
		**N**	**%**		**N**	**%**			
**Gender**									**< 0.001**
	**Female**	**11.535**	**96.9**		**362**	**3.0**			
	**Male**	**4.779**	**95.8**		**206**	**4.1**	**1.4**	**1.1 - 1.6**	
**Age**									**0.527**
	**Under 54 years**	**7.995**	**97.2**		**230**	**2.8**			
	**Above 54 years**	**8.319**	**96.1**		**338**	**3.9**	**1.4**	**1.2 - 1.7**	
**Length of preoperative hospital stay**									**< 0.001**
	**< 24 hours**	**9.657**	**97.8**		**217**	**2.2**			
	**> 24 hours**	**6.657**	**95.0**		**351**	**5.0**	**2.3**	**2.0 - 2.8**	
**Duration of surgery**		**1.6 h**			**2.1 h**		**1.4**	**1.3 - 1.5**	**< 0.001**
**ASA Index** ^**‡**^									
	**I** ^**§**^	**5.317**	**98.0**		**108**	**2.0**			
	**II**	**8.969**	**96.5**		**322**	**3.5**	**1.8**	**1.4 - 2.2**	**< 0.001**
	**III**	**1.812**	**93.6**		**123**	**6.4**	**3.3**	**2.6 - 4.3**	**< 0.001**
	**IV/V**	**216**	**93.5**		**15**	**6.5**	**3.4**	**2.0 - 6.0**	**< 0.001**
**WC** ^**||**^									
	**Clean** ^**§**^	**9.079**	**97.2**		**258**	**2.8**			
	**Cl-Contaminated** ^**¶**^	**5.640**	**96.8**		**187**	**3,2**	**1.2**	**1.0 - 1.4**	**0.114**
	**Contaminated**	**1.266**	**92.8**		**98**	**7.2**	**2.7**	**2.1 - 3.5**	**< 0.001**
	**Dirty/Infected**	**329**	**92.9**		**25**	**7.1**	**2.7**	**1.7 - 4.1**	**< 0.001**
**Emergency surgery**									**< 0.001**
	**No**	**15.494**	**96.8**		**517**	**3.2**			
	**Yes**	**820**	**94.1**		**51**	**5.9**	**1.9**	**1.4 - 2.5**	
**Use of general anesthesia**									**< 0.001**
	**No**	**8.526**	**97.1**		**253**	**2.9**			
	**Yes**	**7.788**	**96.1**		**315**	**3.9**	**1.4**	**1.1 - 1.6**	
**Use of implant**									**0.686**
	**No**	**13.304**	**96.6**		**467**	**3.4**			
	**Yes**	**3.010**	**96.7**		**101**	**3.2**	**0.9**	**0.8 - 1.2**	

* Odds Ratio; † Confidence interval; ‡ *American Society of Anesthesiologists*; § Reference category; || Wound Class; ¶ Clean-Contaminated.

The final model was composed of the following variables (Table 2): length of preoperative hospital stay; duration of surgery; PCSW clean-contaminated, contaminated or dirty/infected and ASA index classified into II, III or IV/V. 

**Table 2:** Final logistic regression model of the independent variables measured in relation to Surgical Site Infection, Belo Horizonte, MG, Brazil, 2011

**Variables**		OR*	**95% CI** ^**†**^	**P value**
**Length of preoperative hospital stay >24 h**		**1.9**	**1.6 - 2.3**	**< 0.001**
**Duration of surgery (in hours)**		**1.3**	**1.3 - 1.4**	**< 0.001**
**PCSW** ^**‡**^				
	**Clean-contaminated**	**1.5**	**1.3 - 1.9**	**< 0.001**
	**Contaminated**	**2.7**	**2.1 - 3.4**	**< 0.001**
	**Dirty/Infected**	**2.0**	**1.3 - 3.2**	**0.001**
**ASA Index** ^**§**^				
	**II**	**1.5**	**1.2 - 1.9**	**<0.001**
	**III**	**2.3**	**1.8 - 3.1**	**< 0.001**
	**IV/V**	**1.9**	**1.1 - 3.4**	**0.031**

* Odds Ratio; † Confidence interval; § *American Society of Anesthesiologists*; ‡ Wound Class.; LLR x^2^ of the final model: 290.61; Pseudo R^2^: 0.0585.

Although the variables gender, general anesthesia and emergency surgery have been selected in the bivariate analysis (p<0.20), to be part of the multivariate analysis, they did not remain in the final logistic model, since they did not reach the level of significance of 5%, previously set for the multivariate analysis.

Of the 568 infections identified, cultures were performed from 177 patients. *Staphyloccocus aureus* (24.3%; 43/177) and *Escherichia coli* (15.3%; 27/177) were the main microorganisms causing SSI.

## Discussion

The overall SSI incidence of 3.4% found in this study was higher than studies carried out in developed countries, such as USA^2^, 1.9%; France, 1.0%^14^ and Italy, 2.6%^15^. However, it was lower than in studies carried out in data reported from India and Turkey, which presented an SSI incidence of 5.0%^16^ ^)^ and 4.1%^17^, respectively. Two Brazilian studies involving SSI in general surgeries had higher rates than the identified incidence and compared to international researches, ranging from 6.4%^8^ to 11.0%^3^. 

The variation in incidence rates observed between the literature and the data from this study may be related to the presence of different epidemiological surveillance systems at national level^2^ ^,^ ^14^ ^-^ ^15^, Post-discharge Surveillance^8^ ^,^ ^16^ (PDS) and possible occurrence of underreporting of SSI.

However, it can be inferred that the low incidence of SSI found in this study may be related to the non-performance of PDS. Data involving orthopedic patients^9^ showed that the non-performance of PDS impacts on the actual SSI rate, which may be 3 times higher when performed only during the hospitalization of the patient.

The risk factors for SSI identified were: length of preoperative hospital stay, duration of surgery, ASA index and PCSW. In this study these risk factors were also identified in researches conducted with a larger number of patients and involving general surgeries^2^ ^,^ ^14^ ^-^ ^17^. In specific surgeries, such as orthopedic surgeries^4^ ^,^ ^6^, factors such as ASA index, Wound class and duration of surgery were statistically associated with SSI, although in head and neck surgeries^5^ and cardiac surgeries^18^, these risk factors have not been identified.

The variable length of preoperative hospital stay greater than 24 hours was associated with approximately twice the chance (OR 1.9) of developing SSI, when compared to a length of hospital stay less than 24 hours (p<0.001). It is important to emphasize that this variable has been found in the literature as a risk factor for SSI in general surgeries^14^ ^-^ ^15^ ^,^ ^17^, but has not been mentioned in other similar studies^18^. In specific surgeries such as orthopedic operations, the length of preoperative hospital stay was not statistically associated with SSI^4^ ^,^ ^6^ ^,^ ^9^.

ANVISA^3^ recommends a preoperative hospital stay of less than 24 hours as an indicator of the process and structure for SSI prevention^3^. A preoperative hospital stay greater than 24 hours is related to a greater incidence of contamination of the patient during the hospitalization period^19^, facilitating the development of infectious processes^20^.

Another variable that showed a statistically significant association with SSI was the duration of surgery. In this study, for each hour of duration of surgery, there was a 34% increase in the chance of SSI development (p<0.001). The duration of surgery is associated with higher SSI rates^4^ ^,^ ^13^ ^-^ ^17^ ^,^ ^21^. It is inferred that this may be related to a greater exposure of the incision site to pathogens^22^ and/or a greater chance of breach of the aseptic technique in the procedure^23^. 

It is worth mentioning that the duration of surgery correlates with other risk factors predisposing to SSI, such as the ASA index, suggesting that patients with higher ASA rates tend to have longer duration of surgery^18^.

 In addition, increased duration of surgery is associated not only with increased SSI rates, but also with other clinical and post-surgical complications such as wound dehiscence, development of Urinary Tract Infection and even septic shock^21^. The search for a shorter duration of surgery can significantly improve the risk of SSI.

The variable PCSW was also statistically associated with SSI. Those surgeries as clean-contaminated, contaminated and dirty/infected showed an increase of 54%, 167% and 105%, respectively, in the chance of developing SSI when compared to clean wounds. The wound class is reported in several nationals and internationals literatures as a risk factor associated with SSI^2^ ^,^ ^6^ ^,^ ^13^ ^-^ ^17^, although it is not present in a research involving general surgeries in Brazil^8^.

In this study, it was found a smaller number of patients classified as dirty/infected wound (354 patients), when compared to the category classified as contaminated (1,364 patients). The reduced risk of SSI in patients classified as infected wound in relation to patients classified as contaminated wound may also be related to the type of intervention adopted for the infected wound, such as the prophylactic antibiotic therapy.

The ASA index for the patient’s clinical status before surgery was statistically associated with SSI. Being classified as ASA II, III and IV/V increases 52%, 134% and 89%, respectively, the chances of developing SSI, when compared to ASA I. Some authors have shown that SSI rates are higher in patients who are more debilitated^24^ or who have systemic diseases, such as Diabetes Mellitus^1^ ^,^ ^18^. Such poorly controlled factors lead to a worsening of the general clinical status of the patient, which implies a higher scoring on the ASA index, making it more susceptible to infections, including SSI. 

The recognition of the ASA index as a risk factor for SSI is observed in different literatures^2^ ^,^ ^4^ ^,^ ^13^ ^-^ ^14^ ^,^ ^17^ ^,^ ^21^. It is important to emphasize that a Brazilian study involving surgeries was identified, which did not use the ASA index for an evaluation of the clinical status of the patient, but rather the presence or absence of pre-existing systemic diseases^8^.

The reduction in the risk of SSI in patients classified as ASA IV/V in relation to ASA III may be related to a lower number of patients classified as ASA IV/V, as observed for the variable Wound class.

The microbiological profile found among the patients who developed SSI was similar to patients that underwent general surgeries, in which *S. aureus* was the main pathogen identified^16^ ^,^ ^25^, but differed from a research carried out in Turkey^17^, which pointed out *E. coli* as the main responsible for the development of SSI and identified in 22.8% of the cases. It is noteworthy that *E. coli* was the second most prevalent microorganism in this study (15.3%).

The identification of risk factors contributes to the creation of SSI prevention strategies, thus allowing health professionals to take actions that reduce complications resulting from infections and minimize SSI rates.

Nursing, as a member of the healthcare team, can carry out specific activities or in collaboration with other professionals to prevent the occurrence of SSI. These activities include: preoperative bath performance^9^ ^,^ ^15^ ^,^ ^18^; better glucose control of the patient diagnosed with Diabetes Mellitus^1^ ^,^ ^18^; control of environmental factors in the operating room^4^ ^,^ ^18^; implementation of PDS protocols^24^, among others.

This study used information from databases, a fact that may limit the accuracy of the results obtained due to the occurrence of information and follow-up biases. The verification of the consistency of the information in each variable of the database and the analysis of the differential loss of the missing data were some strategies used to guarantee the accuracy of the presented results. It should be noted that this study was used a limited number of variables, pre-existing in the hospital database. It is important to note that the non-performance of PDS by HICS may have led to underestimated SSI rates.

## Conclusion

The overall incidence of SSI was 3.4%. The risk factors associated with SSI were: length of preoperative hospital stay greater than 24 hours; a longer duration of surgery; be classified as ASA II, III or IV/V and present PCSW classified as clean- contaminated, contaminated or dirty/infected. Among the SSI cultures analyzed, the most prevalent microorganism was *S. aureus* followed by *E. coli*.

It is important the early recognition of the risk of developing SSI in patients undergoing general surgery, so that preventive measures can be adopted with the aim of reducing infection rates. In this context, new studies using different methodologies and carried out in different circumstances need to be developed in order to add knowledge about SSI in general surgeries.
